# Effect of neonatal nurse mentorship in improving neonatal care competencies among neonatal nurses in Rwandan hospitals

**DOI:** 10.1002/puh2.141

**Published:** 2023-12-21

**Authors:** Marie Louise Manirakiza, Aphrodis Gustave Tuyishime, Amedee Fidele Ndibaza, Francoise Musabeyezu, Benjamin Kulaaza, Francois Biziyaremye, Dieudonne Ndatimana, Richard Kalisa, Diane Rinda, Assumpta Mwali Kayinamura, Christian Mazimpaka

**Affiliations:** ^1^ IntraHealth International Kigali Rwanda

**Keywords:** clinical mentorship, health facilities, neonatal care competencies, nurse

## Abstract

**Background:**

Despite healthcare improvements in Rwanda, newborn mortality remains high. This study assesses the impact of neonatal mentorship on enhancing nurses’ competencies within neonatal units, aiming to address this mortality concern and strengthen healthcare providers’ abilities.

**Methods:**

The prospective cohort study included 25 health facilities supported by Ingobyi Activity in Rwanda, which were beneficiaries of a monthly mentorship program focusing on five critical neonatal competencies. These included adopt manipulation of neonatal equipment, effective management of small and sick newborns, stringent infection prevention and control (IPC), kangaroo mother care (KMC) implementation, and family‐centered care provision. We employed an observation checklist to measure neonatal practice competencies, comparing practices at the time point of the baseline, at the 6th mentorship session, and finally at the 12th mentorship session.

**Results:**

The program engaged 188 neonatal nurse mentees. Data analysis highlighted a substantial increase in overall neonatal practice competencies from a baseline of 42.7%–75.4% after 12 mentorship sessions. Specific competency enhancements included family‐centered care (40.3%–70.3%), IPC (43.2%–84.2%), KMC (56.9%–73.3%), management of small and sick newborns (38.5%–77.6%), and manipulation of neonatal equipment (42.7%–75.4%).

**Conclusions:**

This neonatal mentorship program was effective in enhancing nursing competencies, leading to significant improvements in neonatal care practices. Future work should evaluate the program's cost‐effectiveness and explore its potential to positively impact neonatal health outcomes, thus ensuring sustainable healthcare advancements.

## INTRODUCTION

Globally, the World Health Organization (WHO) has reported a significant concern relating to neonatal mortality, stating that approximately 2.5 million newborns succumb each year within the initial 28 days of life [1]. The stark reality is that more than 98% of these deaths transpire in low‐ and middle‐income countries (LMICs), shedding light on the significant disparities in healthcare outcomes between high‐income and resource‐constrained nations [1]. Rwanda, like many other LMICs, has faced considerable challenges in the realm of neonatal health. Over the years, however, the country has made significant strides to tackle these issues head‐on. The government, in collaboration with various stakeholders, has embarked on a series of interventions with the primary objective of improving neonatal health outcomes [2]. These interventions have revolved around enhancing the accessibility and affordability of healthcare services, increasing the number of healthcare providers, and strengthening their skills through continuous training and mentorship programs. Furthermore, these efforts have sought to broaden the availability and utilization of life‐saving equipment in healthcare facilities across the country [2].

Nevertheless, despite the commendable efforts and the progress made, the neonatal health scenario in Rwanda is far from satisfactory. The country continues to grapple with high neonatal mortality and morbidity rates, especially among premature and low‐birth‐weight infants [3]. These persisting challenges underline the complex nature of neonatal health and the need for comprehensive, multifaceted strategies to improve outcomes. The 2019–2020 Rwanda Demographic and Health Survey offers a sobering snapshot of the stagnant progress in reducing neonatal mortality [4]. The survey results revealed a minute decline in newborn deaths from 20 per 1000 live births in 2015 to 19 per 1000 live births in 2019–2020, indicating that much work remains to be done [4].

The provision of high‐quality health services is significantly hindered by the lack of essential skills and clinical competences among healthcare workers as well as a low coverage of neonatal care services, a predicament that is particularly acute in low‐resource settings [5]. It is common practice to address these competence gaps through training programs. However, these initiatives often occur as isolated events and are typically conducted off‐site, favoring a didactic approach. These trainings, although costly, do not necessarily translate into markedly improved health outcomes. Findings from systematic review done in LMICs show that the often‐used strategies of training only tended to have moderate effects [6]. Compared with training in isolation, effect sizes were generally larger when training was combined with other strategies, such as supervision and group problem solving [6, 7]. An alternative and promising approach to enhance the quality of healthcare services and bolster healthcare providers’ capacity lies in clinical mentorship. This model offers a strategy to supplement traditional training, facilitating the continuous capacity‐building of less seasoned providers through the guidance of their more experienced counterparts. It also ensures that the skills acquired are readily applied to routine clinical practice [8].

The efficacy of clinical mentorship is further underscored by numerous studies that attest to its success in various healthcare areas, including neonatal care. They indicate improvements not only in healthcare providers’ skills and knowledge but also in the overall quality of care [8, 9]. Furthermore, studies found that mentorship's advantages extend to enhancing relationships and collaborations among health providers, influencing attitudes toward work positively, increasing job satisfaction, and boosting confidence among mentees [10].

Recognizing the considerable benefits of mentorship, Ingobyi Activity, an initiative led by IntraHealth International in collaboration with the Ministry of Health in Rwanda, launched a clinical mentorship model. This model aims to strengthen the capacities of neonatal nurses, enabling them to provide higher quality services, thereby helping reduce maternal, newborn, and child mortality and morbidity rates in Rwanda.

This study examined the efficacy of the neonatal nurse mentorship program implemented in 25 hospitals in Rwanda supported by Ingobyi Activity. It primarily assessed the program's impact on improving neonatal nurse mentees’ knowledge and practical skills in newborn health competencies, thus contributing to the broader discourse on capacity building and quality improvement in neonatal care.

## METHODS

### Study setting

This study was carried out in 25 health facilities receiving support from the Ingobyi Activity, which are distributed across 5 provinces of Rwanda [11]. The levels of these facilities were referral hospitals (3), district hospitals (21), and 2 medicalized health centers. The rationale behind the selection of these specific implementation sites was guided by the identified gaps in neonatal care. These gaps were discovered through an initial facility baseline assessment, as well as the findings from supportive supervision activities conducted by staff from the Ingobyi Activity. These assessments helped to locate areas in need of the most urgent intervention and served as the foundation for the targeted mentorship program.

### Study design and population

This was a prospective cohort study conducted in 25 health facility newborn care units among 188 neonatal nurse mentees actively working within these neonatal units. These mentees participated in at least 12 mentorship sessions, facilitated by experienced neonatal nurse mentors.

### Neonatal nurse mentorship intervention

The neonatal nurse mentorship initiative was launched in August 2021. This program was designed to enhance the knowledge and practical skills of neonatal nurses working across various neonatal units in hospitals supported by the project throughout Rwanda. During the initial phase of implementation, 25 neonatal nurses from referral hospitals were purposively selected on the basis of their experience working in neonatology. These nurses further underwent orientation on mentorship principles, supplemented by a refresher course focused on the latest protocols in neonatal care and other pertinent modules they were anticipated to utilize in mentoring their designated mentees.

Subsequently, four to eight neonatal nurses from each hospital were earmarked as mentees. Mentees were selected according to the following criteria: willingness to participate in mentorship program, full time availability, fresh graduate, and being recommended by hospital leadership.

Each mentor was tasked with conducting a 3‐day mentorship visit, the content of which was customized to cater to the unique needs of the mentees in relation to five key competencies. These competencies encompassed the manipulation of neonatal equipment, such as CPAP, syringe/infusion pumps, and infant warmers; management of small and sick newborns; infection prevention and control (IPC) within newborn units; family‐centered care; and kangaroo mother care (KMC).

To gauge the mentees’ absorption and practical application of the knowledge and skills imparted during the mentorship sessions, observation checklists on neonatal care practices were used to see how mentees were performing a task, and then mentors decided on the checklist to be used according to the competence available to perform, knowledge gap, and priority nursing need by the patient.

### Data collection

The process of data collection in this study started from the baseline and continued through to the 12th mentorship session. The progression of the mentees was tracked consistently at every mentorship session with the help of a competency checklist, adapted from the Council of International Neonatal Nurses [12].

Items on the checklist were graded with a score of 3 for appropriate performance, 2 for partial performance, and 1 for missing the task while recognizing some baseline understanding. The score of 1 was not meant to reward noncompliance but to differentiate between complete lack of understanding and basic familiarity. The mentee's total score was the cumulative sum of these codes. This score was then converted to a percentage relative to the maximum expected score.

These scores were subsequently inputted into a Microsoft Excel database and analyzed using Stata software (version 17). A descriptive analysis approach was employed to document the progressive changes observed in each competency from the first session qualified as baseline, sixth session, and twelfth mentorship session. The mentee was qualified as having an appropriate practice if his/her percent score at given competency was at least 80% also termed validated to that competency. The proportion of mentees with appropriate practices was compared at three time points: at the baseline, at the 6th, and at the 12th mentorship session. A one‐way analysis of variance (ANOVA) and a post hoc Tukey test were performed to compare the means of the proportion of mentees with appropriate practice between the baseline, 6th, and 12th mentorship sessions.

### Data analysis

These scores were subsequently inputted into a Microsoft Excel database for analysis then analyzed using Stata software (version 17). A descriptive analysis approach was employed to document the progressive changes observed in each competency from the first session qualified as baseline, sixth session, and twelfth mentorship session. The mentee was qualified as having an appropriate practice if his/her percent score at given competency was at least 80 also termed validated to that competency. The proportion of mentees with appropriate practices was compared at three time points: at the baseline, at the 6th, and at the 12th mentorship session. A one‐way ANOVA and a post hoc Tukey test were performed to compare the means of the proportion of mentees with appropriate practice between the baseline, 6th, and 12th mentorship sessions.

## RESULTS

A total of 4524 neonatal mentorship sessions were conducted across five competencies. Family‐centered care constituted the largest portion of mentorship sessions at 36.1% (1633/4524), followed by small and sick newborn care at 19.2% (868/4524), manipulation of neonatal equipment at 20.6% (931/4524), IPC at 15.6% (708/4524), and KMC at 8.5% (384/4524). The majority (over 80%, or 3769/4524) of mentorship sessions were conducted in district hospitals (Table 1).

**TABLE 1:** Describing neonatal mentorship sessions in different competencies by facility type.

Competencies	Total (*N* = 4524)	
	*N*	%
Family‐centered development care	1633	36.1
Small and sick newborn care	868	19.2
IPC in Neonatal Intensive Care (NICU)	708	15.6
Kangaroo mother care	384	8.5
Use of equipment in newborn unit	931	20.6

Abbreviation: IPC, infection prevention and control.

A significant improvement in the proportion of mentees with appropriate practice was remarked across all competencies from baseline to the 6th visit and up to the 12th visit. The baseline for infant family‐centered development care competency was 40.3% ± 20.7%, rising to 70.3% ± 20.1% by the 12th visit. The small and sick newborn care started at 38.5% ± 19.4% and rose to 77.6% ± 16.9% by the 12th visit. From a baseline of 43.2% ± 21.5%, the IPC in NICU had a substantial increase to 84.2% ± 10.1% by the 12th visit. The KMC competency commenced at 56.9% ± 20.6%, peaked at 76.3% ± 19.4% at the 6th visit, but reduced slightly to 73.3% ± 22.0% by the 12th visit. Finally, the use of equipment in the newborn unit progressed from 44.8% ± 20.6% at baseline to 76.9% ± 17.0% at the 12th visit (Table 2).

**TABLE 2:** Descriptive for proportion of mentees with appropriate practice at different visits across competencies.

	Baseline		At 6th visit		At 12th visit	
	Mean	SD	Mean	SD	Mean	SD
**Infant family–centered development care**	40.3	20.7	64.6	19.2	70.3	20.1
**Small and sick newborn care**	38.5	19.4	69.2	18.0	77.6	16.9
**IPC in NNICU**	43.2	21.5	72.8	14.9	84.2	10.1
**KMC**	56.9	20.6	76.3	19.4	73.3	22.0
**Use of equipment in newborn unit**	44.8	20.6	72.0	18.6	76.9	17.0

Abbreviations: IPC, infection prevention and control; KMC, kangaroo mother care.

Using an ANOVA test, the proportion of mentees' appropriate practices across different neonatal competencies at baseline, the 6th visit, and the 12th visit was evaluated. Results indicated significant differences in mentee practices over the time points for each competency: infant family–centered development care [*F*(2, 385) = 81.2, *p* < 0.001], small and sick newborn care [*F*(2, 202) = 82.4, *p* < 0.001], IPC in NICU [*F*(2, 163) = 73.0, *p* < 0.001], KMC [*F*(2, 87) = 9.6, *p* < 0.001], and use of equipment in the newborn unit [*F*(2, 215) = 58.6, *p* < 0.001] (Table 3).

**TABLE 3:** Analysis of variance (ANOVA) of the proportion of mentees with appropriate practice across different competencies among baseline, 6th visit, and 12th visit.

Competencies	Source of variation	DF	SS	MS	*F*	*p*
**Infant family–centered development care**	Between groups	2	64,702.1	32,351.1	81.2	**0.000**
	Within groups	385	153,402.9	398.4		
	Total	387	218,105.0	563.6		
**Small and sick newborn care**	Between groups	2	56,288.0	28,144.0	82.4	**0.000**
	Within groups	202	68,970.8	341.4		
	Total	204	125,258.8	614.0		
**IPC in NICU**	Between groups	2	45,412.3	22,706.1	73.0	**0.000**
	Within groups	163	50,726.6	311.2		
	Total	165	96,138.9	582.7		
**KMC**	Between groups	2	7832.1	3916.1	9.6	**0.000**
	Within groups	87	35,644.1	409.7		
	Total	89	43,476.2	488.5		
**Use of equipment in newborn unit**	Between groups	2	43,620.0	21,810.0	58.6	**0.000**
	Within groups	215	79,987.8	372.0		
	Total	217	123,607.8	569.6		

Abbreviations: IPC, infection prevention and control; KMC, kangaroo mother care.

Although most competencies saw marked improvements from baseline, the progress between the 6th and 12th visits varied across competencies. Specifically, in “Infant family–centered development care,” there was a significant rise by the 6th visit (mean difference = 24.334, *p* < 0.001) and the 12th visit (mean difference = 30.013, *p* < 0.001) from baseline, yet the incremental progress between 6th and 12th visits was not statistically significant (mean difference = 5.678, *p* = 0.148). Similarly, for “small and sick newborn care,” although there was growth from baseline to both visits, the difference between the 6th and 12th visits was not significant (mean difference = 8.414, *p* = 0.079) (Table 4).

**TABLE 4:** Multiple comparison of dependent variables: proportion of mentees with appropriate practice (Tukey HSD).

Competencies	Visits	Mean difference	Std. error	*t*	Significance	95% CI
**Infant family–centered development care**	Sixth visit vs. baseline	24.334	2.201	11.060	**0.000**	[19.156–29.513]
	Twelfth visit vs. baseline	30.013	3.028	9.910	**0.000**	[22.888–37.137]
	Twelfth visit vs. sixth visit	5.678	3.030	1.870	0.148	[−1.451–12.808]
**Small and sick newborn care**	Sixth visit vs. baseline	30.702	2.802	10.960	**0.000**	[24.086–37.319]
	Twelfth visit vs. baseline	39.116	3.854	10.150	**0.000**	[30.017–48.215]
	Twelfth visit vs. sixth visit	8.414	3.877	2.170	0.079	[−0.740–17.568]
**IPC in NICU**	Sixth visit vs. baseline	29.517	2.962	9.970	**0.000**	[22.511–36.523]
	Twelfth visit vs. baseline	40.926	4.151	9.860	**0.000**	[31.107–50.744]
	Twelfth visit vs. sixth visit	11.409	4.181	2.730	**0.019**	[1.520–21.297]
**KMC**	Sixth visit vs. baseline	19.423	4.584	4.240	**0.000**	[8.493–30.353]
	Twelfth visit vs. baseline	16.474	6.682	2.470	**0.041**	[0.542–32.407]
	Twelfth visit vs. sixth visit	−2.949	6.682	−0.440	0.898	[−18.881–12.984]
**Use of equipment in newborn unit**	Sixth visit vs. baseline	27.229	2.806	9.700	**0.000**	[20.606–33.851]
	Twelfth visit vs. baseline	32.170	4.097	7.850	**0.000**	[22.501–41.840]
	Twelfth visit vs. sixth visit	4.941	4.092	1.210	0.450	[−4.716–14.599]

Abbreviations: IPC, infection prevention and control; KMC, kangaroo mother care.

Table 5 data presents the effect of neonatal nurse mentorship sessions, tracking improvements across a variety of neonatal care practice indicators as the number of mentorship sessions increased. In the category of family‐centered developmental care, we observed significant improvements. The proportion of nurses/midwives maintaining the participation of the family in neonatal care increased from 40.6% at baseline to 66.7% after 6 sessions, and marginally further to 66.8% after 12 sessions. Similar trends are observed in other indicators such as identifying the cultural needs of the family, documenting family care input, and following educational programs for the family (Table 5).

**TABLE 5:** Trend in neonatal care practices indicators by the number of neonatal nurse mentorship sessions.

	Baseline (%)	Six sessions (%)	Twelve sessions (%)
**Family‐centered development care**			
Proportion of nurses/midwives observing strategies that encourage and maintain the participation of the family in planning, delivering, and evaluating neonatal care	40.6	66.7	66.8
Proportion of nurses/midwives observing mentor's use of systems’ knowledge and resources to negotiate optimal continuum of care for the neonate and family	45.8	71.4	86.9
Proportion of nurses/midwives articulating an awareness of stress placed on families and providing appropriate support	43.8	62.5	61.5
Proportion of nurses/midwives developing a high level of sensitivity to identify individuality and the cultural needs of the family	40.1	64.0	70.8
Proportion of nurses/midwives documenting and recording the care input from the family	37.4	63.2	73.1
Proportion of nurses/midwives following educational programs developed to meet the educational needs of the family	34.0	64.9	85.7
Proportion of nurses/midwives recognizing the need for continuity of care to build a professional relationship with family and where possible adhere to the continuity of care	38.0	63.2	70.6
Proportion of nurses/midwives recognizing opportunities for bonding and attachment for the family and neonate	41.4	65.1	70.6
**Infection prevention and control in NICU**			
Proportion of nurses/midwives able to clean equipment correctly on daily basis	37.2	71.5	85.9
Proportion of nurses/midwives able to perform handwashing correctly according to WHO's recommendations	47.4	74.2	88.8
Proportion of nurses/midwives able to perform nursing procedure following aseptic non‐touch technique	45.1	72.7	79.0
**Kangaroo mother care**			
Proportion of nurses/midwives accompanying babies who are transferred in kangaroo mother care room unit	61.7	73.9	77.5
Proportion of nurses/midwives applying kangaroo mother care principles in their neonatal unit	53.5	78.0	69.2
**Small and sick newborn care**			
Proportion of newborns whose intravenous fluids are running appropriately following the rate	38.6	72.3	81.9
Proportion of nurses/midwives able to balance the nutrition and hydration of a sick newborn	36.9	70.6	82.6
Proportion of nurses/midwives able to identify steps needed to do full assessment, triage, and appropriately manage sick and small babies	39.4	68.2	66.3
Proportion of nurses/midwives able to perform advanced neonatal resuscitation	39.2	67.1	78.3
**Use of equipment in newborn unit**			
Proportion of nurses/midwives able to manipulate syringe pumps/infusion pumps	47.8	69.3	74.3
Proportion of nurses/midwives able to manipulate all available equipment in neonatology	42.5	69.9	76.1
Proportion of nurses/midwives able to manipulate to use incubators/radiant warmers	46.6	77.2	83.0
Proportion of nurses/midwives able to perform bubble CPAP machine	42.2	71.7	74.3
**Total**	**42.7**	**69.3**	**75.4**

Abbreviation: WHO, World Health Organization.

Infection control practices saw a substantial increase in the percentage of nurses/midwives who were able to clean equipment correctly, perform proper handwashing according to WHO recommendations, and follow aseptic non‐touch technique. The metrics related to KMC, small and sick newborn care, and use of equipment in the newborn unit also exhibited substantial improvements, with a few exceptions. For instance, in applying KMC principles, there was a decrease from 78.0% after 6 sessions to 69.2% after 12 sessions. Similarly, the proportion of nurses/midwives who were able to identify steps to manage sick and small babies decreased from 68.2% to 66.3% (Table 5).

## DISCUSSION

Our findings revealed that the neonatal nurses’ mentorship model effectively improved the skills and practices of neonatal nurses working in Rwandan hospital neonatal units. The impact of clinical mentorship in increasing practical skills among healthcare professionals has been noted in different studies. A scoping study that reviewed 65 papers on mentorship from different countries, including Rwanda, concluded that mentorship programs had a high potential to strengthen the nursing workforce [13, 14].

The increase in the proportion of healthcare providers who could effectively use newborn equipment by 32.2%points is a notable achievement of the neonatal mentorship model. However, it is important to acknowledge that the shortage of equipment in some health facilities could have lowered the score. Several studies identified insufficient equipment as a significant barrier to optimal neonatal care [15]. In resource‐limited settings, the lack of essential neonatal equipment can lead to a lack of confidence and inadequate use of available equipment by nurses, as highlighted in a study conducted in Ghana [15]. Thus, addressing the equipment gaps is crucial to ensuring that healthcare providers can effectively use available equipment and provide optimal neonatal care (supporting information).

IPC in neonatal units is critical to ensure better health outcomes for neonates. As noted by Lorenzini, effective IPC measures can significantly reduce infant mortality and antibiotic resistance [16]. The results of our study indicate a significant improvement in the proportion of nurses performing well in IPC measures, which increased to almost 100% at the 12th mentorship session. It is worth noting that the recent IPC measures implemented in response to the COVID‐19 pandemic may have contributed to the improvements seen in this study. As healthcare providers have had to adhere more strictly to infection control protocols in the wake of the pandemic, this may have carried over into their work in neonatology. However, it is important to note that the current study did not specifically examine the impact of COVID‐19 on the outcomes of the neonatal mentorship model.

The proportion of healthcare providers performing well in small and sick newborn competency significantly increased by 100% after 1 year of implementation from the baseline. The proportion of healthcare providers who performed well in this competency at the baseline was low, given the increasing number of premature babies, which underscores the importance of this intervention. A study conducted in several African and Asian countries found that healthcare professionals’ lack of knowledge and competencies in managing small and sick newborns was a major cause of inadequate care in facilities [17]. The findings of this study further highlight the potential of clinical mentorship in promoting and enhancing small and sick newborn care in neonatology.

Limitations of our study include the fact that due to staff rotation and turnover, new mentees joined at various mentorship sessions, which might have affected the continuity and overall impact of the mentorship. This could explain the observed decrease in appropriate practices among some mentees in certain competencies. Additionally, the study did not account for the cumulative effect of monthly supportive supervision carried out at the study sites by other non‐mentor supervisors. Additionally, although the mentees knew they were under observation, such a practice aligns with conventional mentorship protocols in our context. Consequently, their conduct most likely provides an authentic depiction of their abilities. Despite these constraints, our findings offer significant insights into neonatal mentorship's potential advantages in enhancing healthcare provider practices, setting the stage for future research, including the correlation between observed improvements and neonatal health outcomes.

## CONCLUSION

The implementation of the neonatal mentorship model in Rwanda has demonstrated a positive and substantial impact on enhancing healthcare providers’ practices in neonatal care. The proportion of neonatal practice competencies observed a significant increase, leaping from a baseline of 42.7% to 75.4% following 12 sessions of the neonatal nurse mentorship program. What is noteworthy is the impressive growth in neonatal competencies seen predominantly within the first half of the 12 sessions, an indication of the program's intensity and the concerted effort invested by both mentors and mentees in improving neonatal practices to achieve early validation. This observation may suggest that the effectiveness of such a program does not necessarily hinge on its duration, an insight that could guide the design of similar interventions in the future.

Notably, the competency that saw the most significant improvement was in IPC measures. It is plausible that the recent surge in IPC measures, primarily in response to the COVID‐19 pandemic, may have contributed to this marked progress in this study (supporting informantion). Looking forward, it would be advantageous to delve into the cost‐effectiveness of the neonatal mentorship model. This exploration would furnish invaluable insights into the feasibility of deploying this model in diverse settings and whether it offers a good return on investment. Additionally, subsequent studies could also assess whether the observed improvements in healthcare provider practices translate into better neonatal health outcomes, thus further validating the model's efficacy.

## AUTHOR CONTRIBUTIONS

*Conceptualization; writing—original draft; methodology; validation; writing—review and editing; project administration; supervision; formal analysis; and investigation*: Marie Louise Manirakiza. *Conceptualization; writing—review and editing; writing—original draft; methodology; and validation*: Aphrodis Gustave Tuyishime. *Conceptualization; writing—original draft; writing—review and editing; methodology; formal analysis; and data curation*: Amedee Fidele Ndibaza. *Conceptualization; writing—original draft; writing—review and editing; methodology*: Francoise Musabeyezu and Benjamin Kulaaza. *Writing—original draft; writing—review and editing; methodology*: Francois Biziyaremye and Diane Rinda. *Writing—original draft; writing—review and editing; formal analysis; and conceptualization*: Dieudonne Ndatimana. *Conceptualization; writing—original draft; writing—review and editing; project administration*: Richard Kalisa. *Conceptualization; writing—original draft; writing—review and editing; methodology; project administration; supervision; and validation*: Assumpta Mwali Kayinamura. *Writing—original draft; writing—review and editing; conceptualization; formal analysis; supervision; project administration; and methodology*: Christian Mazimpaka.

## CONFLICT OF INTEREST STATEMENT

The authors declare that they have no conflicts of interest.

## FUNDING INFORMATION

No funding was received for this research. The study was conducted as an activity of Ingobyi Activity of IntraHealth International in Rwanda.

## ETHICS STATEMENT

All study participants provided signed informed consent in the local language (Kinyarwanda) prior to data collection. We received ethical approval from the Rwanda National Ethics Committee (Kigali, Rwanda, No: 48/RNEC/2022) as well as from IntraHealth International's Institutional Review Board (Chapel Hill, North Carolina, 27517, United States, No: 21006).

## Supporting information

Supporting information

## Data Availability

The datasets for the current study are available from the corresponding author on request.
