# Supplementary material for: Effect of neonatal nurse mentorship in improving neonatal care competencies among neonatal nurses in Rwandan hospitals
Source: Public Health Chall. 2023 Dec 21;2(4):e141. doi: 10.1002/puh2.141 (PMC12039684; doi:10.1002/puh2.141)
Supplement: Supplementary file 1 — Supporting information [file PUH2-2-e141-s001.docx]

#
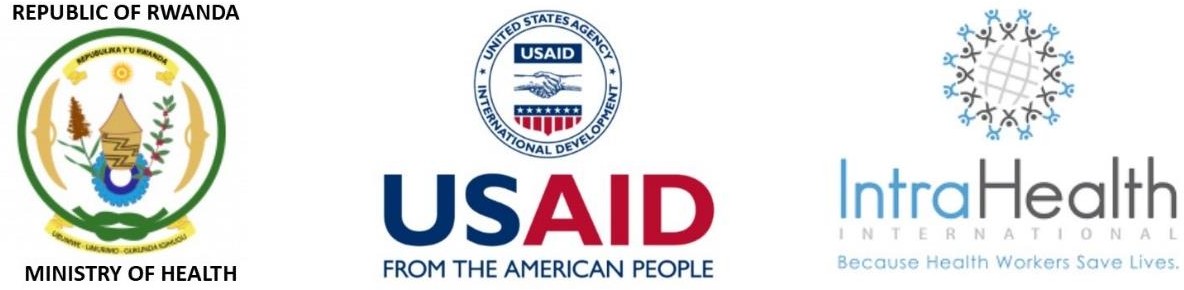


**NEONATAL HEALTH**

**MENTORSHIP ACTIVITY**

NEONATAL COMPETENCY CHECKLIST FRAMEWORK

#

**-**

**MCH AREA 1:** **SMALL AND SICK NEWBRON NURSING CARE**

**Identification**

**District: ……………………………………………………………………**

**Facility name:** …………………………………………………………….

**Name of mentor:** ………………………………………………………….

**Name of mentee:** ………………………………………………………….

**Qualification of mentee:**

A0 Nurse A1 nurse A2 nurse A1 Midwife A0 Midwife Other (specify): ……………………………………………………………

**Instructions:**

Please indicates that the item is done appropriately/ total and write “3” in the box. If the item is done partially write “2” in the box. If the item should have been performed by the mentee but was not, then write “1” in the box.

# Has the mentee attended updated neonatal protocol Training? Yes No

**If yes, when? (**Month/year): ……/…….

| **Visits** | | **1** | **2** | **3** | **4** | **5** | **6** | **7** | **8** | **9** | **10** |
| --- | --- | --- | --- | --- | --- | --- | --- | --- | --- | --- | --- |
| **Dates** | | **----/-----/20…..** | **----/-----/20…..** | **----/-----/20…..** | **----/-----/20…..** | **----/-----/20…..** | **----/-----/20…..** | **----/-----/20…..** | **----/-----/20…..** | **----/-----/20…..** | **----/-----/20…..** |
| **N0** | **Preparation** | | | | | | | | | | |
| 1 | Provider is ready for service delivery |  |  |  |  |  |  |  |  |  |  |
| 2 | Ensure all equipment for evaluation and treatment available |  |  |  |  |  |  |  |  |  |  |
|  | **Initial respiratory system assessment and management of high risk newborn** | | | | | | | | | | |
| 3 | Determine Breathing pattern/rate/WOB (classify mild, moderate and severe respiratory distress) |  |  |  |  |  |  |  |  |  |  |
| 4 | Determine skin colors (types of cyanosis, pallorness and jaundiced ,etc) |  |  |  |  |  |  |  |  |  |  |
| 5 | Secure the airway by ensuring a neutral position using under shoulder roll to maintain neutral position |  |  |  |  |  |  |  |  |  |  |
| 6 | Bulb suction and instill nostrils |  |  |  |  |  |  |  |  |  |  |
| 7 | Machine suction (in case there is a need) |  |  |  |  |  |  |  |  |  |  |
| 8 | Secure and manipulate oxygen tank/concentrator  Pipeline oxygen (where available) |  |  |  |  |  |  |  |  |  |  |
| 9 | Set up and apply nasal cannula in view of respiratory of mild respiratory distress  For newborns ≥ 2 kg or ≥ 33GA  If mild respiratory distress and O2 sat < 90% |  |  |  |  |  |  |  |  |  |  |
| 10 | Set up and apply face mask in case of mild respiratory distress or in case NP is not possible cleft lip  For newborns ≥ 2 kg or ≥ 33GA  If mild respiratory distress and O2 sat < 90% |  |  |  |  |  |  |  |  |  |  |
| 11 | set up and apply nasal bubble CPAP /NCPAP in view of moderate to severe respiratory distress   - every preterm (< 33 GA) and BWT < 2kg - if *any* respiratory distress |  |  |  |  |  |  |  |  |  |  |
| 12 | Set up bag mask |  |  |  |  |  |  |  |  |  |  |
| 13 | Use bag mask on a baby in view of cessation to breathing /apnea and gasping |  |  |  |  |  |  |  |  |  |  |
| 14 | Use pulse oximeter to monitor oxygen saturation after every intervention |  |  |  |  |  |  |  |  |  |  |
| **Initial cardiovascular system assessment and management of high risk newborn** | | | | | | | | | | | |
| 15 | All four-extremity pulse oximetry/blood pressure |  |  |  |  |  |  |  |  |  |  |
| 16 | Placing Cardiac monitor |  |  |  |  |  |  |  |  |  |  |
| 17 | Place pulse oximetry probe |  |  |  |  |  |  |  |  |  |  |
| 18 | Placing cardiac leads |  |  |  |  |  |  |  |  |  |  |
| 19 | Take capirally refill time (press over the sternum for 5 sec and release) (CRF>3 seconds is considered delayed |  |  |  |  |  |  |  |  |  |  |
| 20 | Fill all pulses (central /peripheral |  |  |  |  |  |  |  |  |  |  |
| 21 | Perform Auscultation |  |  |  |  |  |  |  |  |  |  |
| 22 | Heart rate |  |  |  |  |  |  |  |  |  |  |
| 23 | Temperature gradient (extremities temperature and the level of reach) |  |  |  |  |  |  |  |  |  |  |
| 24 | Prepare fluid bolus |  |  |  |  |  |  |  |  |  |  |
| 25 | Determine how much to give in view of signs of poor perfusion(10 ml/kg IV of normal saline over 30 minutes. May be repeated to a maximum of 3 boluses. |  |  |  |  |  |  |  |  |  |  |
| 26 | Perform reassessment immediately after each bolus |  |  |  |  |  |  |  |  |  |  |
| 27 | Determine the needy for IV fluid (If unable to breastfeed or not stable to enterally feed) |  |  |  |  |  |  |  |  |  |  |
| 28 | Check blood glucose if levels |  |  |  |  |  |  |  |  |  |  |
| 29 | Determine the cut off level < 45 mg/dl (2.5 mmol/l) |  |  |  |  |  |  |  |  |  |  |
| 30 | Determine how much and what to give in case of hypoglycemia. |  |  |  |  |  |  |  |  |  |  |
| **Initial neuro system assessment and management of high risk newborn** | | | | | | | | | | | |
| 36 | Assess fontanel |  |  |  |  |  |  |  |  |  |  |
| 37 | Check Mental status |  |  |  |  |  |  |  |  |  |  |
| 38 | Check for motor movement/strength coordination |  |  |  |  |  |  |  |  |  |  |
| 39 | Assess clinical signs of seizures in neonates |  |  |  |  |  |  |  |  |  |  |
| 40 | Use Sarnat staging for assessment of severity in view of HIE per national neonatal protocol |  |  |  |  |  |  |  |  |  |  |

**-**

**-**

|  | **RESULTS OF MENTORSHIP VISIT** | | | | | | | | | | |
| --- | --- | --- | --- | --- | --- | --- | --- | --- | --- | --- | --- |
|  | **Total items evaluated:** |  |  |  |  |  |  |  |  |  |  |
|  | **Mentee total items full completed** |  |  |  |  |  |  |  |  |  |  |
|  | **Percentage achieved (%)** |  |  |  |  |  |  |  |  |  |  |
|  | **Mentor’s signature** |  |  |  |  |  |  |  |  |  |  |
|  | **Mentee’s signature** |  |  |  |  |  |  |  |  |  |  |

**Nutrition and hydration of the sick and small newborn**

| No | **Visits** | **1** | **2** | **3** | **4** | **5** | **6** | **7** | **8** | **9** | **10** |
| --- | --- | --- | --- | --- | --- | --- | --- | --- | --- | --- | --- |
|  | **Dates** | **----/-----/20…..** | **----/-----/20…..** | **----/-----/20…..** | **----/-----/20…..** | **----/-----/20…..** | **----/-----/20…..** | **----/-----/20…..** | **----/-----/20…..** | **----/-----/20…..** | **----/-----/20…..** |
|  | **FOCUSED ASSESSMENT** |  |  |  |  |  |  |  |  |  |  |
| 1 | Vital signs: Temp, HR, RR if possible BP |  |  |  |  |  |  |  |  |  |  |
| 2 | Abdominal bowel sounds/inspection |  |  |  |  |  |  |  |  |  |  |
| 3 | Skin assessment for edema/lesions/distention |  |  |  |  |  |  |  |  |  |  |
| 4 | Intake/Output |  |  |  |  |  |  |  |  |  |  |
| 5 | Nasal gastric tube placement |  |  |  |  |  |  |  |  |  |  |
| 6 | Nutritional status: weight/height |  |  |  |  |  |  |  |  |  |  |
| 7 | determine the appropriate amount of daily fluids the baby needs based on the Rwandan guidelines fluid chart |  |  |  |  |  |  |  |  |  |  |
| 8 | Verbalize the process of weaning from iv fluids |  |  |  |  |  |  |  |  |  |  |
| 9 | Monitor for hypoglycemia |  |  |  |  |  |  |  |  |  |  |
| 10 | Monitor for hyperglycemia |  |  |  |  |  |  |  |  |  |  |
|  | **Equipment/procedure** |  |  |  |  |  |  |  |  |  |  |
| 11 | Place a nasal gastric tube |  |  |  |  |  |  |  |  |  |  |
| 12 | Confirm placement of nasal gastric tube/ when signing in /out and before feeding |  |  |  |  |  |  |  |  |  |  |
| 13 | Perform heel stick |  |  |  |  |  |  |  |  |  |  |
| 14 | Change IV fluids in the presence of hypoglycemia |  |  |  |  |  |  |  |  |  |  |
| 15 | Change IV fluids in the presence of hyperglycemia |  |  |  |  |  |  |  |  |  |  |
| 16 | Assist with cup feeding |  |  |  |  |  |  |  |  |  |  |
| 17 | Assist with breastfeeding |  |  |  |  |  |  |  |  |  |  |
| 18 | Advocate for appropriate formula |  |  |  |  |  |  |  |  |  |  |
| 19 | Write for appropriate fortifier to increase to 0.74 kcal/cc, 0.8 kcal/cc, 1 kcal/cc |  |  |  |  |  |  |  |  |  |  |
| 20 | Calculates the patients total fluids |  |  |  |  |  |  |  |  |  |  |
| 21 | calculates the required rates |  |  |  |  |  |  |  |  |  |  |
| 22 | Urine collection |  |  |  |  |  |  |  |  |  |  |
| 23 | Diaper counts |  |  |  |  |  |  |  |  |  |  |
| 24 | Calculate urine output using the formula (cc/kg/hr |  |  |  |  |  |  |  |  |  |  |
| 25 | Use pediatric giving set to administer the calculated rate |  |  |  |  |  |  |  |  |  |  |
| 26 | Use the standard giving set to administer the calculated rate |  |  |  |  |  |  |  |  |  |  |
| 27 | Mix common fluids used in the neonate population (D10NS, D10RL,D101/4RL) |  |  |  |  |  |  |  |  |  |  |
| 28 | Verbalize the process of weaning from iv fluids |  |  |  |  |  |  |  |  |  |  |
| 29 | verbalize and apply care of newborn on IV Fluids |  |  |  |  |  |  |  |  |  |  |
| 30 | perform a proper charting of the fluid vis a vis input/output balance chart |  |  |  |  |  |  |  |  |  |  |
| 31 | verbalizes parameters indicating optimal feeding in preterm and term babies |  |  |  |  |  |  |  |  |  |  |

**ADVANCED NEONATAL RESUSCITATION**

| **#** | **Visit** | **1** | **2** | **3** | **4** | **5** | **6** | **7** | **8** | **9** | **10** |
| --- | --- | --- | --- | --- | --- | --- | --- | --- | --- | --- | --- |
|  | **Date** | **----/-----/20---** | **---/---/20---** | **----/----/20--** | **----/----/20--** | **----/----/20--** | **----/---/20---** | **----/--_/20--** | **----/----/20--** | **----/----/20--** | **----/----/20--** |
|  | **1.PRE RESUSCITATION PREPARATION** | | | | | | | | | | |
|  | **A. Prevention of hypothermia** | | | | | | | | | | |
| 1 | Close windows and doors before resuscitation to avoid drafts (warm resuscitation) |  |  |  |  |  |  |  |  |  |  |
| 2 | Pre warm the resuscitation area and cover the newborn (warm resuscitation) |  |  |  |  |  |  |  |  |  |  |
|  | **B. Prevention of infections** | | | | | | | | | | |
| 3 | Ensure resuscitation area (radiant warmer) is well cleaned and disinfected |  |  |  |  |  |  |  |  |  |  |
| 4 | Ensure that resuscitation table is well cleaned  and disinfected |  |  |  |  |  |  |  |  |  |  |
| 5 | Washes hands and maintains clean technique |  |  |  |  |  |  |  |  |  |  |
| 6 | Prepare solution for decontamination of materials |  |  |  |  |  |  |  |  |  |  |
|  | **C. Preparation of resuscitation** | | | | | | | | | | |
| 7 | Identifier a helper and makes an emergency plan |  |  |  |  |  |  |  |  |  |  |
| 8 | Ensure the availability of neonatal  resuscitation equipment (Suction device, suction bulb ambu-bag 250, mask No 00,0 and 1), stethoscope, adrenaline, catheters G 24,NS,D5%,D10%,2cc syringe,10cc syringes, Nasogastric tube. |  |  |  |  |  |  |  |  |  |  |
| 9 | Ensure that all equipments are functional |  |  |  |  |  |  |  |  |  |  |
| 10 | Ensure the availability of oxygen to be used if needed |  |  |  |  |  |  |  |  |  |  |
|  | **D. Advanced resuscitation** | | | | | | | | | | |
| 11 | Determine weither this is a term gestation, breathing or crying and tone |  |  |  |  |  |  |  |  |  |  |
| 12 | Warm, clear air way, if necessary, dry and stimulate |  |  |  |  |  |  |  |  |  |  |
| 13 | Assess the breathing status and Check for heat rate (apical pulse in less than 60 second (Preferably by the stethoscope) |  |  |  |  |  |  |  |  |  |  |
| 14 | Start positive pressure ventilation at 40 breath and spo2 monitoring in case heart rate is below 100 bpm and apnea or gasping |  |  |  |  |  |  |  |  |  |  |
| 15 | Reassess heart rate, breathing and chest movement every 30 sec |  |  |  |  |  |  |  |  |  |  |
| 16 | Assess the adequacy of PPV after 30 sec (Chest rise similar to a normal respiratory effort of infant, able to hear air moving in all lobes) |  |  |  |  |  |  |  |  |  |  |
| 17 | Perform Mask readjustment, Reposition airway, suction mouth ,nose ,open mouth, pressure increase and alternative airway (E TT and laryngeal mask) in case heart remains below 100 bpm with no adequate chest rise ,taking corrective PPV |  |  |  |  |  |  |  |  |  |  |
| 18 | Observe the golden minute regarding breathing or start ventilation |  |  |  |  |  |  |  |  |  |  |
| 19 | Determine/considering the cut off HR to initiate chest compression (Chest compressions are initiated if the infant's heart rate remains <60 bpm despite adequate ventilation for 30 seconds) |  |  |  |  |  |  |  |  |  |  |
| 20 | Coordinate effective three chest compressions per 1 ventilation aiming at 90 compressions per 30 breaths in one minute (3:1 ratio) |  |  |  |  |  |  |  |  |  |  |
| 21 | Relax grip on the chest during IPPV (intermittent positive pressure ventilation), and feel for chest movement during ventilation breaths |  |  |  |  |  |  |  |  |  |  |
| 22 | Reassess after 30 sec to 60 sec of initiating chest compression |  |  |  |  |  |  |  |  |  |  |
| 23 | Administer adrenaline(epinephrine) administration (medications are considered when HR remains below 60 beats per minute after adequate 30 seconds of PPV followed by at least 45 to 60 seconds of coordinated effective chest compressions and ventilation) |  |  |  |  |  |  |  |  |  |  |
| 24 | Mix epinephrine from 1:1000 to 1:10000 concentration ( whole one vial 1mg/1ml add up 9cc of normal saline to make 1mg /10cc |  |  |  |  |  |  |  |  |  |  |
| 25 | Determine the recommended dose adrenaline (Use 0.1-0.3mls/kg (0.01-0.03mg/kg) for IV doses and 0.5-1ml/kg (0.05-0.1mg/kg) if giving through ETT). |  |  |  |  |  |  |  |  |  |  |

| 26 | Consider managing hypovolemia and pneumothorax(in case HR remains below 60bpm) |  |  |  |  |  |  |  |  |  |  |
| --- | --- | --- | --- | --- | --- | --- | --- | --- | --- | --- | --- |
| 27 | Articulate special consideration in resuscitation of preterm babies |  |  |  |  |  |  |  |  |  |  |
| **MENTORSHIP RESULTS** | | | | | | | | | | | |

**-**

**-**

|  | **Total items evaluated:** |  |  |  |  |  |  |  |  |  |  |
| --- | --- | --- | --- | --- | --- | --- | --- | --- | --- | --- | --- |
|  | **Mentee total items full completed** |  |  |  |  |  |  |  |  |  |  |
|  | **Percentage achieved (%)** |  |  |  |  |  |  |  |  |  |  |
|  | **Mentor’s signature** |  |  |  |  |  |  |  |  |  |  |
|  | **Mentee’s signature** |  |  |  |  |  |  |  |  |  |  |

**-**

# Visit 1:

- 1. Please provide a brief evaluation of mentee’s strengths

*(Including what skills improved since last evaluation):*

- 1. Please provide recommendations to improve mentee’s practice

*(Mark recommendations agreed upon for next visit)*:

- 1. Please provide examples of information you shared/skills you demonstrated that were aimed at improving the mentee’s practice:

# Visit 2:

1. Please provide a brief evaluation of mentee’s strengths

*(Including what skills improved since last evaluation)*

1. Please provide recommendations to improve mentee’s practice

*(Mark recommendations agreed upon for next visit)*:

1. Please provide examples of information you shared/skills you demonstrated that were aimed at improving the mentee’s practice:

# Visit 3:

1. Please provide a brief evaluation of mentee’s strengths

*(Including what skills improved since last evaluation):*

1. Please provide recommendations to improve mentee’s practice

*(Mark recommendations agreed upon for next visit)*:

1. Please provide examples of information you shared/skills you demonstrated that were aimed at improving the mentee’s practice:

# Visit 4:

1. Please provide a brief evaluation of mentee’s strengths

*(Including what skills improved since last evaluation*

# Visit 5:

1. Please provide a brief evaluation of mentee’s strengths

*(Including what skills improved since last evaluation):*

# MCH:2. MANIPULATION OF BUBBLE CPAP

**Identification**

**District: ……………………………………………………………………**

**Facility name:** …………………………………………………………….

**Name of mentor:** ………………………………………………………….

**Name of mentee:** ………………………………………………………….

**Qualification of mentee:**

A0 Nurse A1 nurse A2 nurse A1 Midwife A0 Midwife Other (specify): ……………………………………………………………

**Instructions:**

Please indicates that the item is done appropriately/ total and write “3” in the box. If the item is done partially write “2” in the box. If the item should have been performed by the mentee but was not, then write “1” in the box.

# Has the mentee attended updated neonatal protocol ? Yes No

**If yes, when? (**Month/year): ……/…….

|  | | |  |  | |  |  | |  |  |  |  |  |  |  |
| --- | --- | --- | --- | --- | --- | --- | --- | --- | --- | --- | --- | --- | --- | --- | --- |
| **#** | | | **dates** |  | **----/---/20…..** | **----/-----/20…..** | | **----/-----/20…..** | **----/-----/20…..** | **----/-----/20…..** | **----/-----/20…..** | **----/-----/20…..** | **----/-----/20…..** | **----/-----/20…..** | **----/-----/20…..** |
|  | | | **Common principle** |  |  |  | |  |  |  |  |  |  |  |  |
| 1. | | | verbalizes common causes of respiratory problems in newborns |  |  |  | |  |  |  |  |  |  |  |  |
| 2 | | | classify respiratory distress in newborns |  |  |  | |  |  |  |  |  |  |  |  |
| 3 | | | verbalizes the management according to classification |  |  |  | |  |  |  |  |  |  |  |  |
| 4 | | | verbalizes a CPAP flow chart algorithm per national neonatal protocol |  |  |  | |  |  |  |  |  |  |  |  |
|  | | | **RESPIRATORY EQUIPMENT** |  |  |  | |  |  |  |  |  |  |  |  |
|  | | | **CPAP (Bubble CPAP)** |  |  |  | |  |  |  |  |  |  |  |  |
|  | | | **Assessment** |  |  |  | |  |  |  |  |  |  |  |  |
| 5 | | | Verbalize the complications associated with using CPAP |  |  |  | |  |  |  |  |  |  |  |  |
| 6 | | | Verbalize what to assess if the CPAP is reading low pressure |  |  |  | |  |  |  |  |  |  |  |  |
| 7 | | | Verbalize the importance of using heated and humidified flow when using CPAP |  |  |  | |  |  |  |  |  |  |  |  |
|  | | | **Equipment/procedure** |  |  |  | |  |  |  |  |  |  |  |  |
| 8 | | | Set up CPAP (attaches hoses, humidifier, add water in humidifier bottle and oxygen/air) |  |  |  | |  |  |  |  |  |  |  |  |
| 9 | | | select the correct age appropriate size bonnet and nasal interface depending the manufacturer |  |  |  | |  |  |  |  |  |  |  |  |
| 10 | | | place the bonnet and mask/prongs on a patient or doll |  |  |  | |  |  |  |  |  |  |  |  |
| 11 | | | Turn on CPAP machine and run a pre use test before use it on baby |  |  |  | |  |  |  |  |  |  |  |  |
| 12 | | | Identifie the oxygen knob and pressure knob |  |  |  | |  |  |  |  |  |  |  |  |
| 14 | | | Program the Baby for example 21 to 100 % of Fraction of inspired oxygen and 5- 8 cm of water of pressure |  |  |  | |  |  |  |  |  |  |  |  |
| 15 | Demonstrate how to set CPAP low and high alarm limits | | |  |  |  | |  |  |  |  |  |  |  |  |
| 16 | | Decrease oxygen to 50%, and demonstrate how to reset the “high oxygen” alarm (press confirm Fi02 button anytime you adjust oxygen) | |  |  |  | |  |  |  |  |  |  |  |  |

|  | **RESULTS OF MENTERSHIP VISIT** | | | | | | | | | | |
| --- | --- | --- | --- | --- | --- | --- | --- | --- | --- | --- | --- |
|  | **Total items evaluated :** |  |  |  |  |  |  |  |  |  |  |
|  | **Mentee total items full completed** |  |  |  |  |  |  |  |  |  |  |
|  | **Percentage achieved (%)** |  |  |  |  |  |  |  |  |  |  |
|  | **Mentor’s signature** |  |  |  |  |  |  |  |  |  |  |
|  | **Mentee’s signature** |  |  |  |  |  |  |  |  |  |  |

**-**

**-**

1.Please provide a brief evaluation of mentee’s strengths

*(Including what skills improved since last evaluation):*

2.Please provide recommendations to improve mentee’s practice

*(Mark recommendations agreed upon for next visit)*:

3.Please provide examples of information you shared/skills you demonstrated that were aimed at improving the mentee’s practice:

# Visit 4:

1.Please provide a brief evaluation of mentee’s strengths

*(Including what skills improved since last evaluation):*

# Visit 5:

1.Please provide a brief evaluation of mentee’s strengths

*(Including what skills improved since last evaluation):*

# Please provide examples of information you shared/skills you demonstrated that were aimed at improving the mentee’s practice

# MCH AREA3 : INFUSION /SYRINGE/ PUMPS AND INCUBATOR /RADIANTS WARMERS MANIPULATION

**Identification**

**District: ……………………………………………………………………**

**Facility name:** …………………………………………………………….

**Name of mentor:** ………………………………………………………….

**Name of mentee:** ………………………………………………………….

**Qualification of mentee:**

A0 Nurse A1 nurse A2 nurse A1 Midwife A0 Midwife Other (specify): ……………………………………………………………

**Instructions:**

Please indicates that the item is done appropriately/ total and write “3” in the box. If the item is done partially write “2” in the box. If the item should have been performed by the mentee but was not, then write “1” in the box.

# Has the mentee attended updated neonatal protocol training? Yes N0

**If yes, when? (**Month/year): ……/…….

| No | **Visits** | **1** | | **2** | **3** | **4** | **5** | **6** | **7** | **8** | **9** | **10** | |
| --- | --- | --- | --- | --- | --- | --- | --- | --- | --- | --- | --- | --- | --- |
|  | **Dates** | **----/-----/20…..** | | **----/-----/20…..** | **----/-----/20…..** | **----/-----/20…..** | **----/-----/20…..** | **----/-----/20…..** | **----/-----/20…..** | **----/-----/20…..** | **----/-----/20…..** | **----/-----/20…..** | |
|  | **Theory:** |  | |  |  |  |  |  |  |  |  |  | |
| 1 | determine the appropriate amount of daily fluids the baby needs based on the Rwandan guidelines fluid chart |  | |  |  |  |  |  |  |  |  |  | |
| 2 | Verbalize the process of weaning from iv fluids |  | |  |  |  |  |  |  |  |  |  | |
| 3 | Calculates the patients total fluids |  | |  |  |  |  |  |  |  |  |  | |
| 4 | calculates the required rates |  | |  |  |  |  |  |  |  |  |  | |
| 5 | Urine collection |  | |  |  |  |  |  |  |  |  |  | |
| 6 | Diaper counts |  | |  |  |  |  |  |  |  |  |  | |
| 7 | Calculate urine output using the formula (cc/kg/hr |  | |  |  |  |  |  |  |  |  |  | |
| 8 | Use pediatric giving set to administer the calculated rate |  | |  |  |  |  |  |  |  |  |  | |
| 9 | Use the standard giving set to administer the calculated rate |  | |  |  |  |  |  |  |  |  |  | |
| 10 | Mix common fluids used in the neonate population (D10NS, D10RL,D101/4RL) |  | |  |  |  |  |  |  |  |  |  | |
| 11 | Verbalize the process of weaning from iv fluids |  | |  |  |  |  |  |  |  |  |  | |
| 12 | verbalize and apply care of newborn on IV Fluids |  | |  |  |  |  |  |  |  |  |  | |
| 13 | perform a proper charting of the fluid vis a vis input/output balance chart |  | |  |  |  |  |  |  |  |  |  | |
| 14 | verbalizes parameters indicating optimal feeding in preterm and term babies |  | |  |  |  |  |  |  |  |  |  | |
|  | **Equipment/procedure** | | | | | | | | | | | | |
|  | **SYRINGE PUMP** |  | |  |  |  |  |  |  |  |  |  | |
| 15 | Performs rate calculations |  | |  |  |  |  |  |  |  |  |  | |
| 16 | Set 7mls to infuse over one hour |  | |  |  |  |  |  |  |  |  |  | |
| 17 | Set 15 mls to infuse over 30 mins |  | |  |  |  |  |  |  |  |  |  | |
| 18 | Set 10 mls to infuse over 3 hours |  | |  |  |  |  |  |  |  |  |  | |
| 19 | Set 3 mls to infuse over 20 minutes |  | |  |  |  |  |  |  |  |  |  | |
|  | **Performance:** |  | |  |  |  |  |  |  |  |  |  | |
| 20 | Turns on syringe pump |  | |  |  |  |  |  |  |  |  |  | |
| 21 | Install a syringe |  | |  |  |  |  |  |  |  |  |  | |
| 22 | Set pump to administer 7mls over one hour (7ml/hr) |  | |  |  |  |  |  |  |  |  |  | |
| 23 | Starts the infusion |  | |  |  |  |  |  |  |  |  |  | |
| 24 | Stop the infusion |  | |  |  |  |  |  |  |  |  |  | |
| 25 | Give a 2ml bolus |  | |  |  |  |  |  |  |  |  |  | |
| 26 | Identifie how to determine the volume that has been infused |  | |  |  |  |  |  |  |  |  |  | |
| 27 | Turns off syringe pump |  | |  |  |  |  |  |  |  |  |  | |
|  | **INFUSION PUMP (Depending on the model)** |  | |  |  |  |  |  |  |  |  |  | |
|  | **Performance:** |  | |  |  |  |  |  |  |  |  |  | |
| 28 | Turn the infusion pump on |  | |  |  |  |  |  |  |  |  |  | |
| 30 | Install the infusion tubing and a syringe into the pump |  | |  |  |  |  |  |  |  |  |  | |
| 31 | Set the pump to infuse 10ml/hr |  | |  |  |  |  |  |  |  |  |  | |
| 31 | Start the infusion |  | |  |  |  |  |  |  |  |  |  | |
| 32 | Stop the infusion |  | |  |  |  |  |  |  |  |  |  | |
| 34 | Identify how to determine the volume that has been infused |  | |  |  |  |  |  |  |  |  |  | |
| 35 | Turn off the infusion pump |  | |  |  |  |  |  |  |  |  |  | |
| **INCUBATORS /RADIANTS WARMERS MANIPULATION** | | | | | | | | | | | | | |
|  | INCUBATORS AND RADIANTS WARMERS | | | | | | | | | | | |  |
| 1 | verbalizes principle of thermoregulation in relation to neonatal energy triangle |  |  | |  |  |  |  |  |  |  | |  |
| 2 | Verbalizes ways of heat loss in newborns |  |  | |  |  |  |  |  |  |  | |  |
| 3 | verbalizes newborns at risk of developing hypothermia |  |  | |  |  |  |  |  |  |  | |  |
| 4 | verbalizes a structured thermoregulation routine care in neonatal unit |  |  | |  |  |  |  |  |  |  | |  |
| 5 | verbalizes IPCs measures with regard to thermoregulation |  |  | |  |  |  |  |  |  |  | |  |
|  | **Theory: use of radiant warmer** | | | | | | | | | | | |  |
| 6 | Verbalizes why a newborn needs to be on a radiant warmer |  |  | |  |  |  |  |  |  |  | |  |
| 7 | Verbalizes why “baby” mode is better then “manual” mode |  |  | |  |  |  |  |  |  |  | |  |
| 8 | Verbalizes all the indication for a radiant warmer |  |  | |  |  |  |  |  |  |  | |  |
| 9 | verbalizes steps for cleaning and Disinfections |  |  | |  |  |  |  |  |  |  | |  |
|  | **Performance on use of radiant warmer** | | | | | | | | | | | |  |
| 11 | Turns on the Radiant warmer |  |  | |  |  |  |  |  |  |  | |  |
| 12 | Changes the mode from pre-heat to baby |  |  | |  |  |  |  |  |  |  | |  |
| 13 | Verbalizes where the temperature probe should be placed |  |  | |  |  |  |  |  |  |  | |  |
| 14 | Sets the temperature at 36.6 degrees Celsius |  |  | |  |  |  |  |  |  |  | |  |
| 15 | Identifies the babies temperature reading |  |  | |  |  |  |  |  |  |  | |  |
| 16 | Turns on examination light |  |  | |  |  |  |  |  |  |  | |  |
| 17 | Identify other useful modality depending on the model |  |  | |  |  |  |  |  |  |  | |  |
| 18 | Supervise/ensure the cleaning and disinfection of a radiant warmer( this can be done by the support staff as well0 |  |  | |  |  |  |  |  |  |  | |  |
|  | **Theory on use incubators** | | | | | | | | | | | |  |
| 19 | Verbalizes why the incubator is better for low birth weight and premature |  |  | |  |  |  |  |  |  |  | |  |
| 20 | Verbalizes how to adjust the incubator if the baby is hypothermic or hyperthermic |  |  | |  |  |  |  |  |  |  | |  |
| 21 | Verbalize the steps of cleaning and disinfection of an incubators | | | | | |  |  |  |  |  | |  |
|  | **Performance on use of incubators** | | | | | | | | | | | |  |
| 22 | Turns on the incubator |  |  | |  |  |  |  |  |  |  | |  |
| 23 | Adjusts the height of the incubator using the foot peddles |  |  | |  |  |  |  |  |  |  | |  |
| 24 | Opens and closes the small doors and the sides of the incubator |  |  | |  |  |  |  |  |  |  | |  |
| 25 | Put the incubator in “skin” mode, and sets the temperature to 36.5 |  |  | |  |  |  |  |  |  |  | |  |
| 26 | Set the temperature to 37.5 (requires pressing the >37 button first) |  |  | |  |  |  |  |  |  |  | |  |

|  | **RESULTS OF MENTERSHIP VISIT** | | | | | | | | | | |
| --- | --- | --- | --- | --- | --- | --- | --- | --- | --- | --- | --- |
|  | **Total items evaluated :** |  |  |  |  |  |  |  |  |  |  |
|  | **Mentee total items full completed** |  |  |  |  |  |  |  |  |  |  |
|  | **Percentage achieved (%)** |  |  |  |  |  |  |  |  |  |  |
|  | **Mentor’s signature** |  |  |  |  |  |  |  |  |  |  |
|  | **Mentee’s signature** |  |  |  |  |  |  |  |  |  |  |

**-**

**-**

**-**

**-**

# Visit 1:

1.Please provide a brief evaluation of mentee’s strengths

*(Including what skills improved since last evaluation):*

2.Please provide recommendations to improve mentee’s practice

*(Mark recommendations agreed upon for next visit)*:

3.Please provide examples of information you shared/skills you demonstrated that were aimed at improving the mentee’s practice:

# Visit 4:Please provide a brief evaluation of mentee’s strengths

*(Including what skills improved since last evaluation):*

# Visit 5:.Please provide a brief evaluation of mentee’s strengths

*(Including what skills improved since last evaluation):*

# MCH AREA 4: INFECTION PREVENTION AND CONTROL IN NEONATAL UNITS

**Identification**

**District: ……………………………………………………………………**

**Facility name:** …………………………………………………………….

**Name of mentor:** ………………………………………………………….

**Name of mentee:** ………………………………………………………….

**Qualification of mentee:**

A0 Nurse A1 nurse A2 nurse A1 Midwife A0 Midwife Other (specify): ……………………………………………………………

**Instructions:**

Please indicates that the item is done appropriately/ total and write “3” in the box. If the item is done partially write “2” in the box. If the item should have been performed by the mentee but was not, then write “1” in the box.

# Has the mentee attended updated neonatal protocol training? Yes N0

**If yes, when? (**Month/year): ……/…

| No | **Visits** | **1** | **2** | **3** | **4** | **5** | **6** | **7** | **8** | **9** | **10** |
| --- | --- | --- | --- | --- | --- | --- | --- | --- | --- | --- | --- |
|  | **Dates** | **----/-----/20…..** | **----/-----/20…..** | **----/-----/20…..** | **----/-----/20…..** | **----/-----/20…..** | **----/-----/20…..** | **----/-----/20…..** | **----/-----/20…..** | **----/-----/20…..** | **----/-----/20…..** |
| I | **perform handwashing correctly according to WHO recommendation** |  |  |  |  |  |  |  |  |  |  |
| 1 | Perform hand washing before touching the newborn |  |  |  |  |  |  |  |  |  |  |
| 2 | Perform hand washing after touching the newborn |  |  |  |  |  |  |  |  |  |  |
| 3 | Perform hand washing before procedure |  |  |  |  |  |  |  |  |  |  |
| 4 | Perform hand washing after procedure |  |  |  |  |  |  |  |  |  |  |
| 5 | Perform hand washing after exposure to bodily fluid |  |  |  |  |  |  |  |  |  |  |
| 6 | Avail alcohol-based hand rub (not less than 60% of alcohol is not accepted in neonatology the recommendation is to do handwashing ) |  |  |  |  |  |  |  |  |  |  |
| II | **clean equipment correctly on daily basis** |  |  |  |  |  |  |  |  |  |  |
| 1 | clean daily with soap and water and decontaminated using a 0.5% chlorine ,making sure that all visible stains are wiped off.( this may be done at each shift if necessary, but making sure that there is a change of the cleaning materials between incubators) |  |  |  |  |  |  |  |  |  |  |
|  | Easily contaminates all monitor probes and cuffs in-between patients’ use, therefore need for daily cleaning with detergent and with disinfectant. |  |  |  |  |  |  |  |  |  |  |
| 2 | Clean CPAP circuits and soak them in 0.5%chlorine bleach for 15-20min before being reinsing them |  |  |  |  |  |  |  |  |  |  |
| 3 | Wash and disinfect reusable equipment before application to patient |  |  |  |  |  |  |  |  |  |  |
| III | **perform nursing procedure following aseptic non-touch technique** |  |  |  |  |  |  |  |  |  |  |
| 1 | Wear clean gloves for all handlings of invasive devices such as peripheral IV’s that are indwelling |  |  |  |  |  |  |  |  |  |  |
| 2 | Provide skin antisepsis prior to invasive procedures such as cannulation using the appropriate antiseptic options |  |  |  |  |  |  |  |  |  |  |
| 3 | discontinue invasive devices as soon as possible when it is appropriate |  |  |  |  |  |  |  |  |  |  |
| 4 | handle neonates with confirmed sepsis with precautions (respecting IPC measures) in order to not spread infection to other patient and environment |  |  |  |  |  |  |  |  |  |  |
| 5 | Keep arms bare to the elbows during direct patient care. |  |  |  |  |  |  |  |  |  |  |

|  | **RESULTS OF MENTERSHIP VISIT** | | | | | | | | | | |
| --- | --- | --- | --- | --- | --- | --- | --- | --- | --- | --- | --- |
|  | **Total items evaluated :** |  |  |  |  |  |  |  |  |  |  |
|  | **Mentee total items full completed** |  |  |  |  |  |  |  |  |  |  |
|  | **Percentage achieved (%)** |  |  |  |  |  |  |  |  |  |  |
|  | **Mentor’s signature** |  |  |  |  |  |  |  |  |  |  |
|  | **Mentee’s signature** |  |  |  |  |  |  |  |  |  |  |

**-**

**-**

# Visit 1:

1.Please provide a brief evaluation of mentee’s strengths

*(Including what skills improved since last evaluation):*

2.Please provide recommendations to improve mentee’s practice

*(Mark recommendations agreed upon for next visit)*:

3.Please provide examples of information you shared/skills you demonstrated that were aimed at improving the mentee’s practice:

# Visit 4:

1.Please provide a brief evaluation of mentee’s strengths

*(Including what skills improved since last evaluation):*

2..Please provide recommendations to improve mentee’s practice

*(Mark recommendations agreed upon for next visit)*:

3.Please provide examples of information you shared/skills you demonstrated that were aimed at improving the mentee’s practice:

# Visit 5:

1.Please provide a brief evaluation of mentee’s strengths

*(Including what skills improved since last evaluation):*

2.Please provide recommendations to improve mentee’s practice

*(Mark recommendations agreed upon for next visit)*:

3.Please provide examples of information you shared/skills you demonstrated that were aimed at improving the mentee’s practice:

**-**

**-**

**MCH:5.** **FAMILY CENTERED DEVELOPMENTAL SUPPORT AND KANGAROU MATHER CRE**

**Instructions:**

Please indicates that the item is done appropriately/ total and write “3” in the box. If the item is done partially write “2” in the box. If the item should have been performed by the mentee but was not, then write “1” in the box.

# Has the mentee updated neonatal protocol training ? Yes No

**If yes, when? (**Month/year): ……/…….

|  | **Visits** | **1** | **2** | **3** | **4** | **5** | **6** | **7** | **8** | **9** | **10** |
| --- | --- | --- | --- | --- | --- | --- | --- | --- | --- | --- | --- |
|  | **Dates** | **----/-----/20…..** | **----/-----/20…..** | **----/-----/20…..** | **----/-----/20…..** | **----/-----/20…..** | **----/-----/20…..** | **----/-----/20…..** | **----/-----/20…..** | **----/-----/20…..** | **----/-----/20…..** |
|  | **Assessment** |  |  |  |  |  |  |  |  |  |  |
| I | **Observe strategies that encourage and maintain the participation of the family in the planning, delivering, and evaluating neonatal care** |  |  |  |  |  |  |  |  |  |  |
| 1 | Deliver relevant information to parents |  |  |  |  |  |  |  |  |  |  |
| 2 | encourage family to attend the ward round if applicable |  |  |  |  |  |  |  |  |  |  |
| 3 | Engage parents to do some small task for their babies(feeding,positioning) |  |  |  |  |  |  |  |  |  |  |
| II | **Observe mentor’s use of systems’ knowledge and resources to negotiate optimal continuum of care for the neonate and family •** |  |  |  |  |  |  |  |  |  |  |
| 1 | advocates for the neonate and their family's needs |  |  |  |  |  |  |  |  |  |  |
| 2 | coordinate the care for a premature neonate and providing support to the baby's family |  |  |  |  |  |  |  |  |  |  |
| 3 | learns how to provide emotional support to the family |  |  |  |  |  |  |  |  |  |  |
| III | **Articulate an awareness of stress placed on families and providing appropriate support** |  |  |  |  |  |  |  |  |  |  |
| 1 | actively listens to their concerns and emotions. |  |  |  |  |  |  |  |  |  |  |
| 2 | Recognizing the importance of cultural competence |  |  |  |  |  |  |  |  |  |  |
| 3 | schedules regular check-ins with the family to assess their evolving needs and provide ongoing emotional support |  |  |  |  |  |  |  |  |  |  |
| IV | D**evelop a high level of sensitivity to identify individuality and the cultural needs of the family** |  |  |  |  |  |  |  |  |  |  |
| 1 | uses open-ended questions to encourage families to share their cultural beliefs and practices |  |  |  |  |  |  |  |  |  |  |
| 2 | approaches each family with a deep sense of respect and a non-judgmental attitude |  |  |  |  |  |  |  |  |  |  |
| 3 | actively seeks feedback from families to ensure that their cultural needs are being met |  |  |  |  |  |  |  |  |  |  |
|  | **Follow educational programs developed to meet the educational needs of the family** |  |  |  |  |  |  |  |  |  |  |
| 1 | follow any educational program targeting family centered |  |  |  |  |  |  |  |  |  |  |
| 2 | Assess the educational needs of the family |  |  |  |  |  |  |  |  |  |  |
| 3 | schedules educational sessions at times that are convenient for the family |  |  |  |  |  |  |  |  |  |  |
|  | **Recognize opportunities for bonding and attachment for the family and neonate** |  |  |  |  |  |  |  |  |  |  |
| 1 | prioritize on encouraging the family to stay a bit with the baby is not part of the bonding and attachment because it disrupt the nursing care activities |  |  |  |  |  |  |  |  |  |  |
| 2 | Allow the parents to touch their babies |  |  |  |  |  |  |  |  |  |  |
| VII | **Document and recording the care input from the family** |  |  |  |  |  |  |  |  |  |  |
| 1 | takes notes on the family's input. This may include their concerns about the child's symptoms, preferences for care routines, dietary restrictions, or requests for specific comfort measures |  |  |  |  |  |  |  |  |  |  |
| 2 | discusses the family's input with the healthcare team and incorporates relevant suggestions into the child's care plan |  |  |  |  |  |  |  |  |  |  |
| 3 | ensures that all family-related input is handled with the utmost privacy and confidentiality, in compliance with healthcare regulations and guidelines |  |  |  |  |  |  |  |  |  |  |
| **KANGAROU MATHER CARE** | | | | | | | | | | | |
| I | **Accompany babies who are transferred in kangaroo mother care room unit** |  |  |  |  |  |  |  |  |  |  |
| 1 | Assemble all equipment need for KMC for neonatal transport |  |  |  |  |  |  |  |  |  |  |
| 2 | Ensures that the mother is able to visualize /sense the babies breathing |  |  |  |  |  |  |  |  |  |  |
| II | **Apply kangaroo mother care principles in their neonatal unit** |  |  |  |  |  |  |  |  |  |  |
| 1 | Provide all stable babies in NN U with at least one hour of KMC(Intermittent KMC) Daily |  |  |  |  |  |  |  |  |  |  |
| 2 | Ensure that Babies in KMC room receives 18-20hours of KMC daily |  |  |  |  |  |  |  |  |  |  |
|  | **Total items evaluated :** |  |  |  |  |  |  |  |  |  |  |
|  | **Mentee total items full completed** |  |  |  |  |  |  |  |  |  |  |
|  | **Percentage achieved (%)** |  |  |  |  |  |  |  |  |  |  |
|  | **Mentor’s signature** |  |  |  |  |  |  |  |  |  |  |
|  | **Mentee’s signature** |  |  |  |  |  |  |  |  |  |  |

# Visit 1:

1.Please provide a brief evaluation of mentee’s strengths

*(Including what skills improved since last evaluation):*

2.Please provide recommendations to improve mentee’s practice

*(Mark recommendations agreed upon for next visit)*:

3.Please provide examples of information you shared/skills you demonstrated that were aimed at improving the mentee’s practice:

# Visit 2:

1.Please provide a brief evaluation of mentee’s strengths

*(Including what skills improved since last evaluation)*

2.Please provide recommendations to improve mentee’s practice

*(Mark recommendations agreed upon for next visit)*:

3.Please provide examples of information you shared/skills you demonstrated that were aimed at improving the mentee’s practice:

# Visit 3:

1.Please provide a brief evaluation of mentee’s strengths

*(Including what skills improved since last evaluation):*

2.Please provide recommendations to improve mentee’s practice

*(Mark recommendations agreed upon for next visit)*:

3.Please provide examples of information you shared/skills you demonstrated that were aimed at improving the mentee’s practice:

# Visit 4:

1.Please provide a brief evaluation of mentee’s strengths

*(Including what skills improved since last evaluation):*

2.Please provide recommendations to improve mentee’s practice

*(Mark recommendations agreed upon for next visit)*:

3.Please provide examples of information you shared/skills you demonstrated that were aimed at improving the mentee’s practice:

# Visit 5:

1.Please provide a brief evaluation of mentee’s strengths

*(Including what skills improved since last evaluation):*

2.Please provide recommendations to improve mentee’s practice

*(Mark recommendations agreed upon for next visit)*:

3.Please provide examples of information you shared/skills you demonstrated that were aimed at improving the mentee’s practice:
